# Effects of low-temperature stress during rice heading stage on carbon and nitrogen allocation in paddy eco-system of northeastern China

**DOI:** 10.3389/fpls.2025.1484734

**Published:** 2025-03-21

**Authors:** Tao Sun, Junmei Ruan, Tiehua Cao, Li Yao, Zichao Zhao, Jun Zhang, Jiarui Li, Aixing Deng, Haotian Chen, Xinhao Gao, Zhenwei Song

**Affiliations:** ^1^ State Key Laboratory of Nutrient Use and Management/Institute of Agricultural Resources and Environment, Shandong Academy of Agricultural Sciences, Jinan, China; ^2^ Key Laboratory of Wastes Matrix Utilization, Ministry of Agriculture and Rural Affairs/Institute of Agricultural Resources and Environment, Shandong Academy of Agricultural Sciences, Jinan, China; ^3^ Key Laboratory of Crop Physiology and Ecology, Ministry of Agriculture and Rural Affairs/Institute of Crop Sciences, Chinese Academy of Agricultural Sciences, Beijing, China; ^4^ Institute of Grain and Oil, Meizhou Academy of Agriculture and Forestry Sciences, Meizhou, China; ^5^ Institute of Agricultural Resources and Environment, Jilin Academy of Agricultural Sciences, Changchun, China

**Keywords:** rice, chilly stress, carbon and nitrogen partition, isotope labelling, high latitude area

## Abstract

**Introduction:**

In high-latitude area, climate change has brought about recurrent chilling stress that adversely impacts the sustainable production of rice and alters the distribution of carbon (C) and nitrogen (N) in paddy ecosystems. A comprehensive understanding of how the paddy ecosystem’s C and N allocation responds to low-temperature stress during critical growth stages remains elusive.

**Methods:**

A rice pot experiment of two varieties combined with ^13^C and ^15^N isotope labelling method was conducted to evaluate how low temperature stress at heading stage affects rice yield, and above- and belowground C and N partitioning.

**Results and Discussion:**

Low-temperature stress significantly reduced rice grain yield of JN809 (sensitive to low-temperature stress) and J88 (tolerant to low-temperature stress) varieties by 27.6% and 21.4%, respectively, This stress tendency increased C and N accumulation in rice stems and leaves, while concurrently decreasing C and N accumulation in panicles. Specifically, under low-temperature stress, the ^13^C isotope content in stems and leaves was found to be 14.0% and 19.0% higher than in the control treatment, while the ^13^C and ^15^N isotope contents in their panicles were 29.3% and 22.5% lower, respectively. The low-temperature tolerant variety (J88) demonstrated a reduced effect of low-temperature stress on rice yield and C, N allocation due to efficient resource reallocation and stress tolerance mechanisms. The findings of this study provide a foundation for developing rice breeding and cultivation techniques that can enhance rice resilience and adaptability to climate change. Additionally, it informs strategies to optimize C and N sequestration practices in rice fields, ensuring high yields and efficient resource utilization.

## 1 Introduction

Since the beginning of twenty-first century, the frequency and intensity of agricultural meteorological disasters have been on the rise due to climate change (Palmer, 2014; Ju et al., 2013; Chen et al., 2020). Meanwhile, the Fifth Assessment Report of the Intergovernmental Panel on Climate Change (IPCC_AR5) pointed out that as global climate warming and extreme weather events become more frequent, the risk of low-temperature stress may also increase, which to a certain extent increases the instability of agricultural production (Pachauri et al., 2014). Addressing agricultural meteorological disasters is urgent to maintain sustainable crop production (Gomez-Zavaglia et al., 2022).

Rice (*Oryza sativa* L.) is the most important staple food for more than half of the population across the world. It was estimated that global rice demand would increase from 644 million tons in 2007 to 827 million tons in 2050 (Alexandratos and Bruinsma, 2012). As the world’s largest producer and consumer, China plays a central role in meeting this increasing demand. Along with global warming, the rice cropping area in northeast China has increased rapidly, now accounting for more than 14% of China’s total production (National Bureau of Statistics of China, NBSC, 2023). Even more, nearly 75% of rice production in northeast China was for commodities. In recent years, extreme temperature events have occurred frequently in rice planting areas of China (Wang et al., 2019; Yuan et al., 2021), and cold damage has become the primary meteorological disaster affecting rice production, particularly in northeast China (Tao et al., 2013). Low-temperature stress is one of the major limiting factors affecting stable rice production in high-latitude areas (e.g. northeast China). Delayed-type or obstructive-type cold damage seriously affects rice growth, development, and grain filling (Tao et al., 2013). However, current research mainly focuses on the characteristics and mechanisms of the expected impact of rising atmospheric temperatures on C and N distribution in rice field systems (Tang et al., 2021, Tang et al., 2022; Haas et al., 2022; Xie et al., 2023), while extreme low-temperatures also affect rice growth and lead to differences in the distribution of C and N between rice crops, soil, and atmosphere (Deng et al., 2011).

Rice has a specific minimum suitable temperature at each growth and development stage, low-temperature stress is unfavorable for dry matter accumulation. Especially during the heading stage, rice exhibits its weakest tolerance to low-temperatures. Cold stress during this period can lead to floret degradation or pollen sterility, resulting in empty grains (Zhang et al., 2017; Siddik et al., 2019), reducing grain setting rate and thousand-grain weight, ultimately causing a significant decline in yield (Zhu et al., 2012; Sperotto et al., 2013). Furthermore, different varieties had varying tolerances to low-temperature stress (Wainaina et al., 2015; Geraldine et al., 2020). Low-temperature stress caused a certain degree of damage to the antioxidant system of flag leaves in rice. When the temperature was too low, the activity of protective enzymes in rice leaves was affected, leading to the impairment of rice photosynthesis and other functions, which in turn affected the stable formation of the final yield (Gazquez et al., 2018). At present, research on rice low-temperature damage mainly focuses on the degree of low-temperature damage, changes in rice physiological functions during low-temperatures, and the impact on yield components and quality (Bhattacharjees, 2013; Ren et al., 2017). However, there are limited reports on the effects of low-temperature damage and rice varieties on rice, particularly concerning the distribution of C and N and changes in soil C and N. Given the strong C sink function and outstanding C sequestration potential of the paddy ecosystems (Liu et al., 2023), understanding how low-temperature stress during critical growth stages and rice varieties affects C and N dynamics is crucial for addressing climate change.

Hereby, we hypothesized that (i) the biomass and yield of rice were affected by low-temperature during the rice heading stage; (ii) low-temperature significantly hindered the transportation of C assimilate and absorbed N from soil resulting in the re-distribution of C and N in paddy ecosystem. To test these hypotheses, an artificial climate chamber combined with a pot experiment and ^13^C and ^15^N isotope labeling methods were used to simulate extreme low-temperature conditions in the paddy ecosystem. The objectives of the present study are to evaluate the effects of low-temperature stress during the rice heading stage on C and N distribution in aboveground and underground parts of rice crops and changes in soil C and N in soils. It is anticipated to provide a basis for rice breeding and cultivation techniques that can enhance rice resilience and adaptability to climate change, as well as optimize C and N sequestration practices in the paddy ecosystem to ensure high yields and resilience to climate disaster risks.

## 2 Materials and methods

### 2.1 Experimental design

The experiment was conducted in Gongzhuling City, Jilin Province of China (124°48´E, 43°32´N) in 2019. Two-factor experiment with randomized block design was used to evaluate how air temperature stress and different rice varieties affect C and N allocation in above-ground and under-ground parts of rice, the changes in C and N in rhizosphere soil and bulk soil. The temperature treatments included the control treatment (CK, no low-temperature stress) and low-temperature stress treatment at the heading stage (LT). The rice varieties included Jijing 88 (J88, super japonica rice tolerant to low-temperature stress) and Jinongda 809 (JN809, sensitive to low-temperature stress) (https://www.ricedata.cn/; Zhang et al., 2021). The experiment adopted the pot planting method. There were sixteen pots for each treatment and a total of 64 pots for the experiment. Four pots, for each treatment and sampling date, were used for parameter measurement at the rice heading stage (5^th^ August), grain filling stage (27^th^ August), and harvesting stage (23^rd^ September). The pot was 25 cm in height with a diameter of 20 cm. A nylon bag with a height of 10 cm and a diameter of 8 cm was buried in the basin as the root bag. The mesh size of the nylon bag is 37 μm, which ensures that the rice roots do not extend out of the root bag, but can ensure the exchange of nutrients between the root bag and soil in the pot. The total amount of soil in the basin and the root bag was 5.5 kg. The potting soil was a paddy soil developed from long-term conventional paddy fields and formed by red clay in quaternary soil. The soil is characterized as soil organic carbon of 21.3 g kg^-1^, total N of 2.0 g kg^-1^, alkali-hydrolyzed N of 176.1 mg kg^-1^, available phosphorus of 19.4 mg kg^-1^, available potassium of 64.1 mg kg^-1^, and pH of 5.6. Three uniformly growing rice seedlings (Rice with four leaves and one heart) are transplanted in each root bag at the seedling stage, maintaining a 3-4 cm water layer, and fertilized and managed according to conventional local practices, with N at 0.276 g kg^-1^, P_2_O_5_ at 0.272 g kg^-1^, and K_2_O at 0.185 g kg^-1^. The N fertilizer is urea (CO(NH_2_)_2_, containing 46% N), which is applied three times in the ratio of 3:4:3 as base fertilizer, tillering fertilizer, and grain filling fertilizer respectively. The P fertilizer was (NH_4_)_2_HPO_4_ (containing 53% P_2_O_5_), and the K fertilizer was K_2_SO_4_ (containing 54% K_2_O). Both P_2_O_5_ and K_2_O fertilizers are applied as base fertilizers at one time. The rice was grown under natural conditions except for the heading stage when low-temperature stress was conducted.

### 2.2 ^13^C and ^15^N labelling

In order to differentiate the allocation of C and N assimilation in rice system, ^13^C and ^15^N isotope labelling methods were used in the present experiment. ^13^C marker is started at the booting stage. For labelling, rice was confined in a transparent labelling chamber (1 m length × 1 m width × 1 m height for each chamber, **Supplementary Figure S1A**) and after half an hour of confinement treatment, H_2_SO_4_ was dripped into ^13^C-labelled Na_2_CO_3_ (^13^C abundance >98%, Shanghai Research Institute of Chemical Technology, Shanghai, China) to produce ^13^CO_2_, which was aired after 4 hours of confinement and labelled continuously for 7 days (**Supplementary Figure S1A**). The ^15^N labelling was carried out simultaneously with ^13^C labelling by adding ^15^NH_4_Cl (^15^N abundance 99.9%, Shanghai Research Institute of Chemical Technology, Shanghai, China) in pots. The labelling amounts of ^13^C and ^15^N are 56.3 mg ^13^C pot^-1^ and 40.8 mg ^15^N pot^-1^, respectively. The control treatment applied the same amount of unlabeled NH_4_Cl. Fans and ice packs were placed inside for physical cooling during the marking period to prevent high temperatures in the labelling chamber.

### 2.3 Low-temperature stress simulation

**Figure 1:**
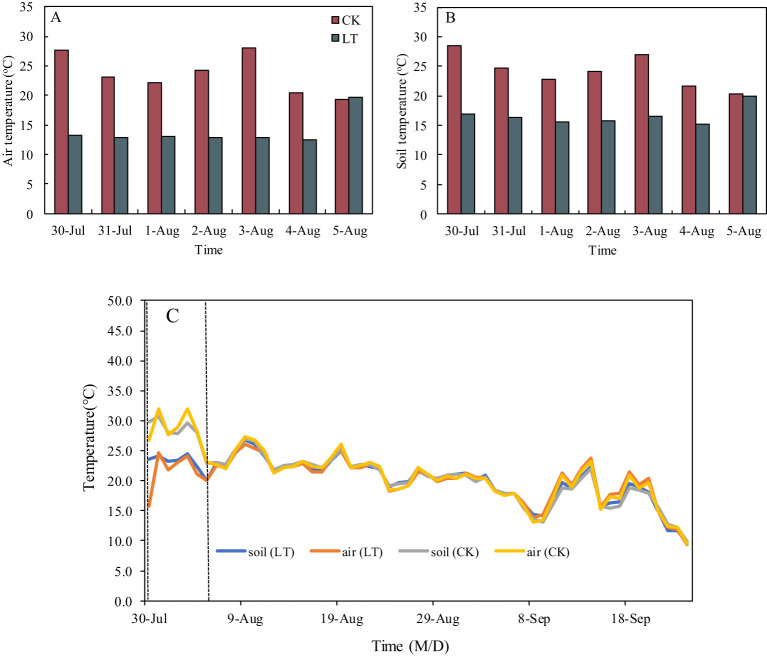
Air **(A)** and soil **(B)** temperature during low-temperature treatment between CK and LT treatments, air and soil temperature **(C)** during the experiment period between CK and LT treatments. CK and LT denote control treatment and low-temperature treatment, respectively.

An artificial climate chamber was used to simulate low-temperature stress at the rice heading stage. After the ^13^C and ^15^N labelling, half of the pots for each rice variety were moved into the artificial climate chamber (**Supplementary Figure S1B, C**), and the low-temperature treatment was conducted from 20: 00 pm to 8: 00 am the next day. The extreme low-temperature treatment was divided into four periods of 3 hours each, and the temperature of each period from the beginning to the end was 14°C, 11°C, 10°C and 13°C in order. The temperature of the control treatment from the beginning to the end was 25°C, 23°C, 22°C and 24°C in order (**Supplementary Figure S1D**). The artificial climate chamber was controlled to give potted plants light at 5:00 a.m. The extreme low-temperature treatment lasted for 7 days (from the night of July 30 to the morning of August 5). **Figure 1** presents the effect of low-temperature treatment on air and soil temperatures. The mean air and soil temperatures from 20:00 to 8:00 under low-temperature treatment were 12.4~13.2°C, and 15.2~16.8 °C, respectively, which was suitable for simulating chilling stress in the experimental site. After the low-temperature treating completed, all the pots were moved from the artificial climate chamber to the natural conditions. After the low-temperature treatment, there were no differences in soil and air temperatures among all the treatments (**Figure 1C**).

### 2.4 Plant and soil sampling and measurement

Plant and soil samples were collected three times at the rice heading stage (5^th^ August, 2019), the grain filling stage (27^th^ August, 2019), and the harvesting stage (23^rd^ September, 2019). Four pots were collected at each sampling. The rice was pulled out with the root and the rhizosphere soil was collected by shaking method when sampling. The bulk soil was also sampled from the pot. The rice plants were separated according to the root, stem, leaf, and panicle, then put into the oven at 105°C for 30 min to kill, and then baked at 65°C until constant weight. After taking out, the dry matter weight of each part was weighed on a one-hundredth scale. The rhizosphere and bulk soil were placed in a cool and dry environment to dry naturally. The soil and plant samples were ground with a ball mill until they met the test criteria. The processed samples were determined by isotope mass spectrometry (Elementar Isoprime 100, Germany) to determine the values of total C and N as well as δ^13^C and δ^15^N for each fraction.

The contents of ^13^C and ^15^N in plants (root, stem, leaf and panicle) and soil (rhizosphere and bulk soils) can be calculated by the following formula (Equations 1, 2).


(1) $${\;}^{13}{\text{C}}_{\text{i}}={\text{C}}_{\text{i}}\times ({\text{δ}}^{13}{\text{C}}_{\text{i}}-{\text{δ}}^{13}{\text{C}}_{\text{n}})/100\times 1000$$

(2) $${\;}^{15}{\text{N}}_{\text{i}}={\text{C}}_{\text{i}}\times ({\text{δ}}^{15}{\text{N}}_{\text{i}}-{\text{δ}}^{15}{\text{N}}_{\text{n}})/100\times 1000$$

^13^C_i_ and ^15^N_i_ represent ^13^C and ^15^N contents (mg· pot^-1^), δ^13^C_i_ and ^13^C_n_ represent abundance of ^13^C in labeled and unlabeled samples (‰), δ^15^N_i_ and ^15^N_n_ represent abundance of ^15^N in labeled and unlabeled samples (‰), respectively. C_i_ and N_i_ represent the total C and N content (mg· pot^-1^) in plants (panicle, stem, leaf and root) and soil (rhizosphere and bulk soil).

The total assimilation of ^13^C and ^15^N was the sum of ^13^C and ^15^N of plants (root, stem, leaf, panicle) and soils (rhizosphere soil, bulk soil) measured at the end of labeling (Equations 3, 4).


(3) $$\begin{array}{c} {\text{Total}}^{13}\text{C assimilation}\\ {=}^{13}\text{C in panicle }{+}^{13}\text{C in stem}{+}^{13}\text{C in leaf}{+}^{13}\text{C in root}\\ {+}^{13}\text{C in rhizosphere soil}{+}^{13}\text{C in bulk soil} \end{array}$$

(4) $$\begin{array}{c} {\text{Total}}^{15}\text{N assimilation}\\ {=}^{15}\text{N in panicle}{+}^{15}\text{N in stem}{+}^{15}\text{N in leaf}{+}^{15}\text{N in root}\\ {+}^{15}\text{N in rhizosphere soil }{+}^{15}\text{N in bulk soil} \end{array}$$

The incorporation of ^13^C (% of assimilated ^13^C) and ^15^N (% of assimilated ^15^N) into panicle, stem, leaf, root, rhizosphere, and bulk soil at each sampling date were calculated as follows (Equations 5, 6):


(5) $${\;}^{13}\text{C incorporation }\left(\%\right){=}^{13}{\text{C}}_{i}/{\text{Total}}^{13}\text{C assimilation}\times 100\%$$

(6) $${\;}^{15}\text{N incorporation }\left(\%\right){=}^{15}{\text{N}}_{i}/{\text{Total}}^{15}\text{N assimilation}\times 100\%$$

### 2.5 Statistical analysis

The PROC GLM procedure in SAS (SAS Institute Inc., Version 9.2, Cary, NC) was used to analyze the effects of treatments on rice biomass, rice yield and its component factors, ^15^N and ^13^C contents, percentage distribution of the assimilated ^15^N and ^13^C. The parameters mentioned above were analyzed using the MIXED procedure of SAS, with sampling date, temperature treatment, and variety used as fixed effects Littell et al, 2006, and Compound symmetry covariance structure was used for repeated measures analysis. Means were compared using Fisher’s protected least significant difference (LSD) test at the 0.05 probability level. To assess the linear relationship between rice yield and the C^13^/N^15^ ratio, Pearson correlation analysis was employed.

## 3 Results

### 3.1 Rice biomass

**Figure 2:**
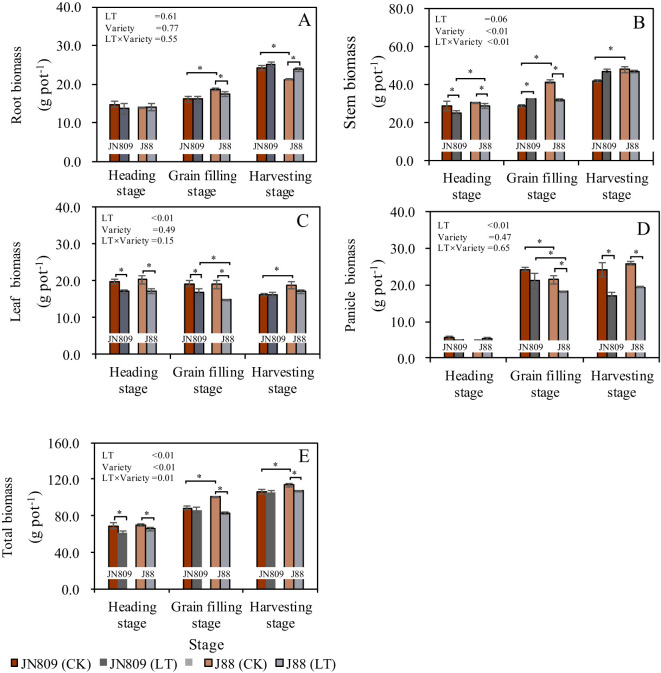
Root **(A)**, stem **(B)**, leaf **(C)**, panicle **(D)** and total biomass **(E)** during different growing stages. CK and LT denote control treatment and low-temperature treatment, respectively. Bar means the standard error. “*” indicate significant differences between the CK and LT treatments, and between different varieties at the probability level of 0.05.

Low-temperature treatment during the rice heading stage and different rice variety treatments both influenced dry matter accumulation and distribution across various growth stages (**Figure 2**). Low-temperature treatment generally reduced total dry matter accumulation in rice plants, whereas rice varieties affected leaf and panicle biomass. At the grain-filling stage, low-temperature treatment decreased root biomass of J88 by 7.2% compared to the control (CK). At the heading stage, stem biomass decreased by 12.5% in JN809 and by 6.5% in J88. However, at the grain-filling stage, stem biomass in JN809 increased by 12.3%. Low-temperature treatment reduced leaf biomass in both rice varieties. Leaf biomass decreased by 13.7% in JN809 and by 15.4% in J88 at the heading stage, and by 12.8% in JN809 and 22.1% in J88 at the grain-filling stage. Panicle biomass of J88 under low-temperature treatment at the grain-filling stage was 15.3% lower than CK. At the harvesting stage, panicle biomass under low-temperature treatment was 29.0% lower in JN809 and 25.0% lower in J88 compared to CK. At the heading stage, low-temperature treatment decreased total biomass by 11.6% in JN809 and by 6.5% in J88. At the grain-filling stage, total biomass in J88 decreased by 18.2%. At the harvesting stage, total biomass in J88 decreased by 8.4%.

### 3.2 Rice yield and its component factors

**Figure 3:**
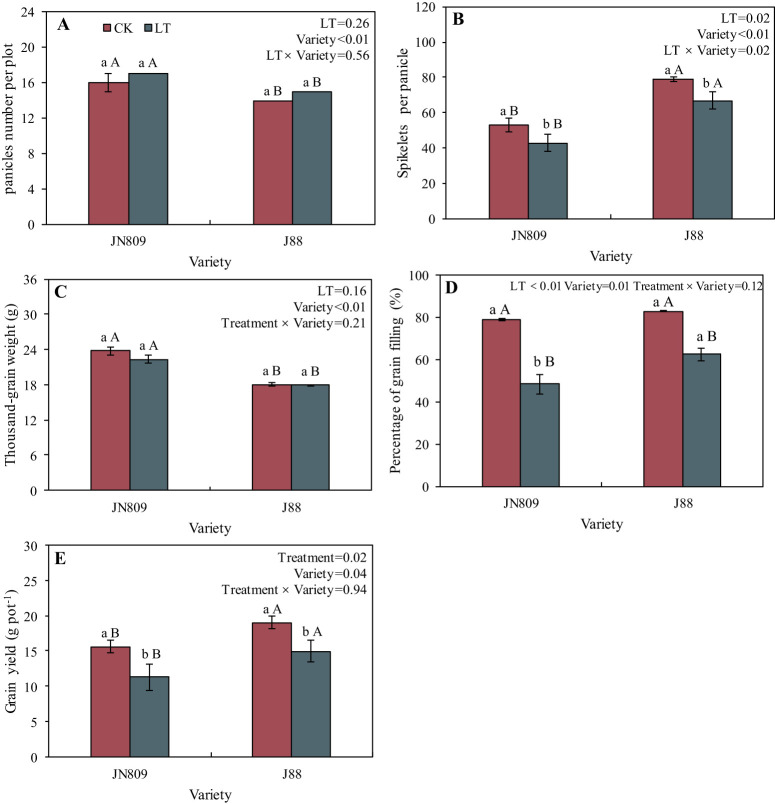
Grain yield and its component factors under different temperature treatments and varieties. **(A)** Panicle number per plot, **(B)** Spikelets per panicle, **(C)** Thousand-grain weight, **(D)** Percentage of grain filling, **(E)** Grain yield). CK and LT denote control treatment and low-temperature treatment, respectively. Bar means the standard error. Different lowercase letters for the same variety indicate significant differences between the CK and LT treatments at the probability level of 0.05, while different capital letters for the same temperature treatment indicate significant differences between different varieties at the probability level of 0.05.

The low-temperature treatment impacted spikelets per panicle, percentage of grain filling and yield (**Figure 3**). While the rice panicles number per plot, spikelets per panicle, thousand-grain weight, percentage of grain filling and yield varied between varieties. The number of spikelets per panicle under low-temperature treatment was 20.2% and 15.1% lower in JN809 and J88 than in CK, respectively. The rice yield of JN809 and J88 under low-temperature treatment was 27.6% and 21.4% lower than those of CK, respectively. The lower-temperature treatment decreased the percentage of grain filling of JN809 and J88 by 38.5% and 24.8%, when compared with the CK treatment. The low-temperature treatment had no effect on the number of panicles and thousand-grain weight of rice. The spikelets per panicle and yield of JN809 were 57.4% and 32.3% lower than those of the J88 variety, respectively.

### 3.3 N uptake

**Figure 4:**
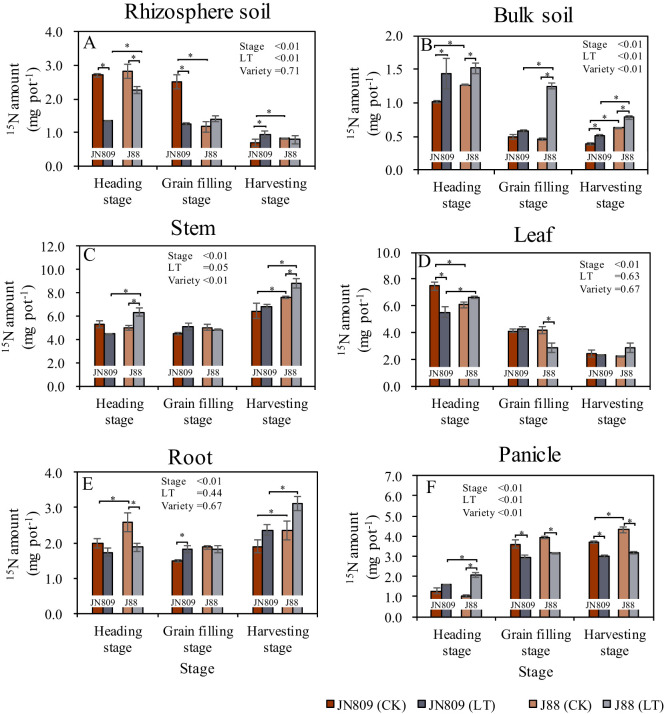
The content of ^15^N retained in rice-soil system during different growing stages (Unit: mg pot^-1^). **(A)** Rhizosphere soil, **(B)** Bulk soil, **(C)** Stem, **(D)** Leaf, **(E)** Root, **(F)** Panicle). CK and LT denote control treatment and low-temperature treatment, respectively. Bar means the standard error. “*” indicate significant differences between the CK and LT treatments, and between different varieties at the probability level of 0.05.

The sampling period, low-temperature treatment, and rice variety all impacted nitrogen (N) uptake by rice (**Figure 4**). Low-temperature treatment reduced rhizosphere soil ^15^N content. At the heading stage, rhizosphere soil ^15^N content of JN809 and J88 under low-temperature was 50.1% and 20.0% lower than CK treatment, respectively. At the grain-filling stage, JN809’s rhizosphere soil ^15^N content under low-temperature was 49.7% lower than CK. However, at the harvesting stage, JN809’s rhizosphere soil ^15^N content under low-temperature was 36.2% higher than CK. Under low-temperature, J88’s rhizosphere soil ^15^N content was 66.7% higher than JN809 at the heading stage (**Figure 4A**). Low-temperature treatment increased bulk soil ^15^N content. At the heading stage, bulk soil ^15^N content under low-temperature was 41.4% and 19.9% higher in JN809 and J88, respectively, compared to CK. At the grain-filling stage, J88’s bulk soil ^15^N content under low-temperature was 173.7% higher than CK. At the harvesting stage, bulk soil ^15^N content under low-temperature was 30.5% and 24.4% higher in JN809 and J88, respectively, compared to CK. Under low-temperature, J88’s bulk soil ^15^N content was 115.5% higher at the grain-filling stage and 56.0% higher at the harvesting stage, compared to JN809 (**Figure 4B**). Low-temperature treatment also increased stem ^15^N content. At the heading stage, J88’s stem ^15^N content under low-temperature was 26.5% higher than CK. At the harvesting stage, J88’s stem ^15^N content under low-temperature was 15.5% higher than CK. Compared to JN809, J88’s stem ^15^N content under low-temperature was 42.2% higher at the heading stage, and 28.2% higher at the harvesting stage (**Figure 4C**).

Low-temperature treatment reduced leaf ^15^N content. At the heading stage, leaf ^15^N content of JN809 under low-temperature treatment was 26.6% lower than CK, while for J88, it was 32.3% lower. Under low-temperature, leaf ^15^N content of J88 was 20.5% higher than JN809 at the heading stage (**Figure 4D**). Root ^15^N content under low-temperature treatment was 27.5% lower in J88 at the heading stage, compared to CK, but 22.4% higher in JN809. At the harvesting stage, root ^15^N content of J88 under low-temperature was 32.2% higher than JN809 (**Figure 4E**). Panicle ^15^N content under low-temperature treatment was 103.4% higher in J88 at the heading stage, compared to CK. However, it was 18.0% lower in JN809 and 18.9% lower in J88 at the grain-filling stage. At the harvesting stage, panicle ^15^N content was 18.8% lower in JN809 and 26.1% lower in J88, compared to CK. Under low-temperature, panicle ^15^N content of J88 was 27.2% higher than JN809at the heading stage (**Figure 4F**).

### 3.4 C uptake

**Figure 5:**
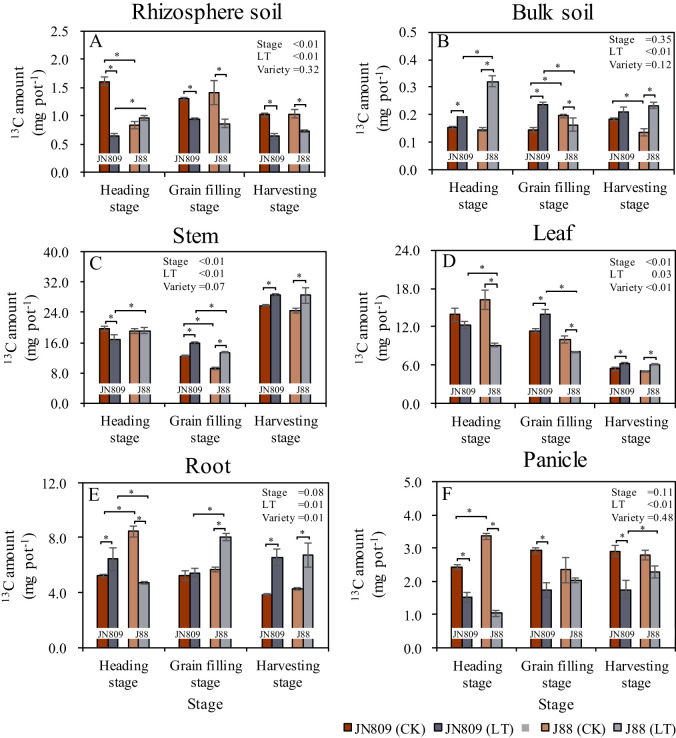
The content of ^13^C retained in the rice-soil system (Unit: mg pot^-1^). **(A)** Rhizosphere soil, **(B)** Bulk soil, **(C)** Stem, **(D)** Leaf, **(E)** Root, **(F)** Panicle). CK and LT denote control treatment and low-temperature treatment, respectively. Bar means the standard error. “*” indicate significant differences between the CK and LT treatments, and between different varieties at the probability level of 0.05.

The sampling period, low-temperature treatment, and rice variety all influenced the partitioning of photosynthetic products in rice (**Figure 5**). At the heading stage, rhizosphere soil ^13^C content under low-temperature treatment was 60.1% lower in JN809, compared to CK. At the grain-filling stage, it was 27.5% lower in JN809 and 38.9% lower in J88. At the harvesting stage, it was 37.8% lower in JN809 and 29.5% lower in J88 compared to CK. Under low-temperature, rhizosphere soil ^13^C content of J88 was 50.0% higher than JN809 at the heading stage (**Figure 5A**). Low-temperature treatment increased bulk soil ^13^C content. At the heading stage, bulk soil ^13^C content under low-temperature treatment was 25.8% higher in JN809 and 118.5% higher in J88, compared to CK. At the grain-filling stage, it was 62.1% higher in JN809, and at the harvesting stage, it was 72.6% higher in J88, compared to CK. Under low-temperature, bulk soil ^13^C content of J88 was 68.4% higher at the heading stage, but 30.4% lower at the grain-filling stage, compared to JN809 (**Figure 5B**). Stem ^13^C content under low-temperature treatment was 13.4% lower in JN809 at the heading stage. At the grain-filling stage, it was 27.4% higher in JN809 and 45.5% higher in J88. At the harvesting stage, it was 10.6% higher in JN809 and 17.3% higher in J88, compared to CK. Under low-temperature, leaf ^13^C content of J88 was 13.1% higher at the heading stage, but 15.4% lower at the harvesting stage, compared to JN809 (**Figure 5C**).

Leaf ^13^C content under low-temperature treatment was 43.6% lower in J88 at the heading stage, 23.2% higher in JN809 at the grain-filling stage, and 16.3% higher in JN809 and 21.6% higher in J88 at the harvesting stage, compared to CK. Under low-temperature, leaf ^13^C content of J88 was 25.2% lower at the heading stage, and 41.9% lower at the grain-filling stage, compared to JN809 (**Figure 5D**). Root ^13^C content under low-temperature treatment was 23.0% higher in JN809 at the heading stage, 41.6% lower in J88 at the grain-filling stage, and 70.1% lower in JN809 and 56.7% lower in J88 at the harvesting stage, compared to CK. Under low-temperature, root ^13^C content of J88 was 26.7% lower at the heading stage, but 47.5% higher at the grain-filling stage, compared to JN809 (**Figure 5E**). Low-temperature treatment decreased panicle ^13^C content. At the heading stage, panicle ^13^C content under low-temperature treatment was 37.9% lower in JN809, and 69.2% lower in J88, compared to CK. At the grain-filling stage, it was 40.8% lower in JN809, and at the harvesting stage, it was 39.9% lower in JN809, compared to CK. Under low-temperature, panicle ^13^C content of J88 was 26.7% higher at the harvesting stage, compared to JN809 (**Figure 5F**).

### 3.5 Variation of the assimilated ^15^N in the rice-soil system

**Figure 6:**
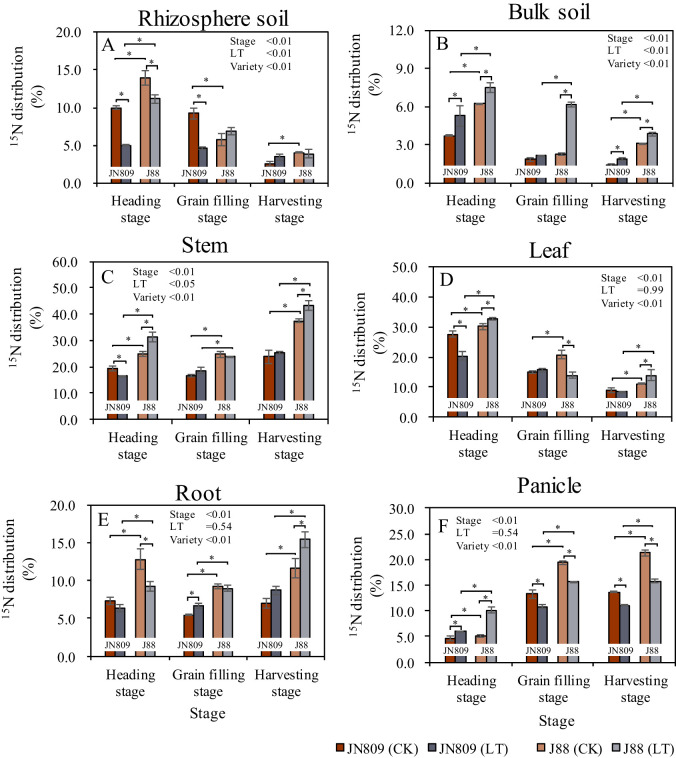
The percentage distribution of ^15^N (content at the end of labeling) in different parts of rice during different growth stages in rice-soil system (Unit: %). **(A)** Rhizosphere soil, **(B)** Bulk soil, **(C)** Stem, **(D)** Leaf, **(E)** Root, **(F)** Panicle). CK and LT denote control treatment and low-temperature treatment, respectively. Bar means the standard error. “*” indicate significant differences between the CK and LT treatments, and between different varieties at the probability level of 0.05.

The sampling period, low-temperature treatment, and rice variety all affected the proportion of ^15^N partitioning (**Figure 6**). Under low-temperature treatment, the percentage distribution of soil ^15^N in the rice rhizosphere soil was 4.99 and 2.79 percentage points lower in JN809 and J88 at the heading stage, respectively, and 4.60 percentage points lower in JN809 at the grain-filling stage, compared to CK. At the heading stage, the percentage distribution of soil ^15^N in the rhizosphere soil of J88 was 6.17 percentage points higher than that of JN809 under low-temperature conditions (**Figure 6A**). Low-temperature treatment increased the percentage distribution of soil ^15^N in the rice bulk soil. At the heading stage, this distribution was 1.55 and 1.24 percentage points higher in JN809 and J88, respectively, compared to CK. At the grain-filling stage, it was 3.91 percentage points higher in J88, and at the harvesting stage, it was 0.43 and 0.76 percentage points higher in JN809 and J88, respectively, compared to CK. Under low-temperature conditions, the percentage distribution of soil ^15^N in the bulk soil of J88 was 2.20 percentage points higher at the heading stage, 4.04 percentage points higher at the grain-filling stage, and 2.01 percentage points higher at the harvesting stage compared to JN809 (**Figure 6B**). The percentage distribution of soil ^15^N in the rice stem also increased under low-temperature treatment. At the heading stage, it was 6.55 percentage points higher in J88 compared to CK, and at the harvesting stage, it was 5.82 percentage points higher in J88, compared to CK. Under low-temperature conditions, the percentage distribution of soil ^15^N in the stem of J88 was 14.86 percentage points higher at the heading stage, 5.13 percentage points higher at the grain-filling stage, and 18.11 percentage points higher at the harvesting stage, compared to JN809 (**Figure 6C**).

Under low-temperature treatment, the percentage distribution of soil ^15^N in rice leaves was 7.35 percentage points lower in JN809 and 6.72 percentage points lower in J88 at the heading stage, compared to CK. However, at the harvesting stage, this distribution was 2.83 percentage points higher in J88 than CK. Additionally, the percentage distribution of soil ^15^N in the leaves of J88 was 12.46 percentage points higher at the heading stage, and 5.42 percentage points higher at the harvesting stage, compared to JN809 (**Figure 6D**).

In terms of rice roots, the percentage distribution of soil ^15^N under low-temperature treatment was 1.22 percentage points higher in JN809 at the grain-filling stage, and 3.79 percentage points higher in J88 at the harvesting stage, compared to CK. For the J88 variety, this distribution was 2.91 percentage points higher at the heading stage, 2.31 percentage points higher at the grain-filling stage, and 6.70 percentage points higher at the harvesting stage, compared to JN809 (**Figure 6E**). Regarding rice panicles, the percentage distribution of soil ^15^N under low-temperature treatment was 2.38 and 3.67 percentage points lower in JN809 and J88, respectively, at the grain-filling stage, compared to CK. However, at the harvesting stage, it was 2.56 percentage points higher in JN809, and 5.56 percentage points higher in J88, compared to CK. Under low-temperature conditions, the percentage distribution of soil ^15^N in the panicles of J88 was 4.20 percentage points higher at the heading stage, 4.84 percentage points higher at the grain-filling stage, and 4.64 percentage points higher at the harvesting stage compared to JN809 (**Figure 6F**).

### 3.6 Variation of the assimilated ^13^C in the rice-soil system

**Figure 7:**
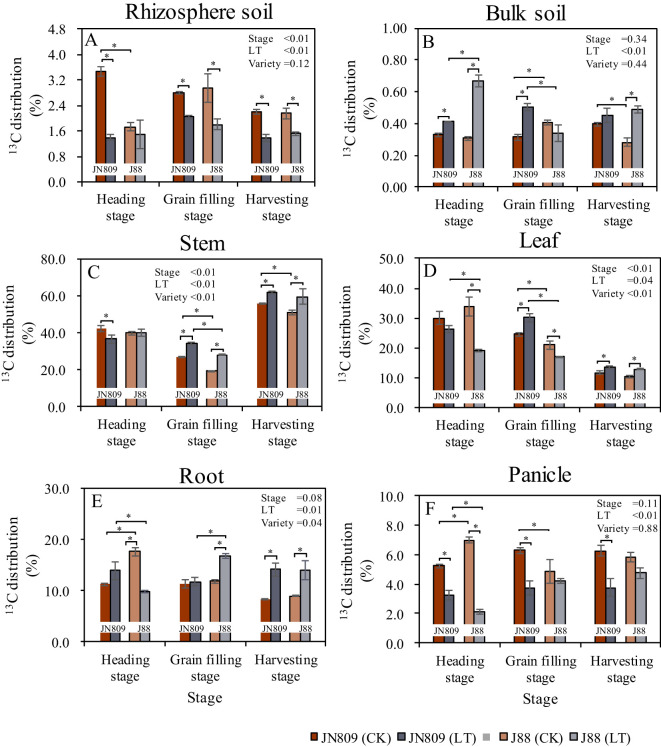
The percentage distribution of ^13^C (content at the end of labeling) in different parts of rice during different growth stages in rice-soil system (Unit: %). **(A)** Rhizosphere soil, **(B)** Bulk soil, **(C)** Stem, **(D)** Leaf, **(E)** Root, **(F)** Panicle). CK and LT denote control treatment and low-temperature treatment, respectively. Bar means the standard error. “*” indicate significant differences between the CK and LT treatments, and between different varieties at the probability level of 0.05.

The sampling period, low-temperature treatment, and rice variety all influenced the allocation of photosynthetic products in rice (**Figure 7**). Low-temperature treatment reduced the percentage distribution of ^13^C in rice rhizosphere soil. At the heading stage, this distribution was 2.09 percentage points lower in JN809 compared to CK. At the grain-filling stage, it was 0.77 percentage points lower in JN809 and 1.17 percentage points lower in J88. At the harvesting stage, it was 0.83 percentage points lower in JN809 and 0.63 percentage points lower in J88, compared to CK (**Figure 7A**). In rice bulk soil, the percentage distribution of ^13^C was 0.08 and 0.36 percentage points higher in JN809 and J88, respectively, at the heading stage compared to CK. At the grain-filling stage, it was 0.19 percentage points lower in JN809, and at the harvesting stage, it was 0.21 percentage points lower in J88 compared to CK. Under low-temperature conditions, the percentage distribution of ^13^C in the bulk soil of J88 was 0.17 percentage points lower at the grain-filling stage, compared to JN809 (**Figure 7B**). For rice stems, the percentage distribution of ^13^C was 7.35 and 8.74 percentage points higher in JN809 and J88, respectively, at the grain-filling stage compared to CK. At the harvesting stage, it was 5.93 percentage points higher in JN809 and 8.80 percentage points higher in J88 compared to CK. Under low-temperature conditions, the percentage distribution of ^13^C in the stems of J88 was 6.19 percentage points lower at the grain-filling stage, compared to JN809 (**Figure 7C**).

In rice leaves, the percentage distribution of ^13^C was 14.73 percentage points lower in J88 at the heading stage, and 3.93 percentage points lower at the grain-filling stage, compared to CK. At the harvesting stage, this distribution was 1.90 percentage points higher in JN809, and 2.27 percentage points higher in J88, compared to CK. Under low-temperature conditions, the percentage distribution of ^13^C in rice leaves was 7.29 percentage points lower in J88 at the grain-filling stage, and 13.22 percentage points lower at the harvesting stage, compared to JN809 (**Figure 7D**). In rice roots, the percentage distribution of ^13^C was 7.80 percentage points lower in J88 at the heading stage, and 4.92 percentage points higher at the grain-filling stage, compared to CK. At the harvesting stage, it was 5.80 percentage points higher in JN809 and 5.07 percentage points higher in J88, compared to CK. Under low-temperature conditions, the percentage distribution of ^13^C in rice roots was 4.05 percentage points lower in J88 at the heading stage, and 5.01 percentage points higher at the grain-filling stage, compared to JN809 (**Figure 7E**). In rice panicles, the percentage distribution of ^13^C was 1.98 and 4.84 percentage points lower in JN809 and J88, respectively, at the heading stage compared to CK. At the grain-filling stage, it was 2.57 percentage points lower in JN809, and at the harvesting stage, it was 2.50 percentage points lower in JN809 compared to CK. Under low-temperature conditions, the percentage distribution of ^13^C in rice panicles was 1.10 percentage points lower in J88 at the heading stage, compared to JN809 (**Figure 7F**).

### 3.7 The relationship between the C^13^/N^15^ ratio and yield

**Figure 8:**
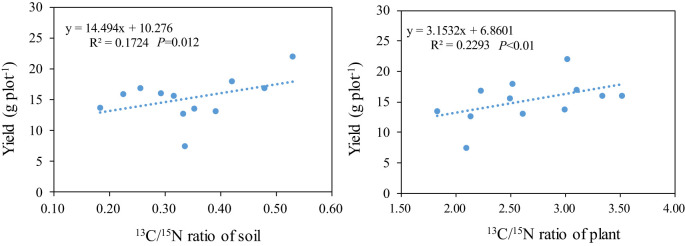
The relationship between the C^13^/N^15^ ratio and yield.

The research revealed a significant positive correlation between the soil’s C^13^/N^15^ ratio and rice yield. As the C^13^/N^15^ ratio in rice plants increased, the rice yield correspondingly rose. This trend was consistent: with higher C^13^/N^15^ ratios in the rice plants, the rice yield also increased (**Figure 8**).

## 4 Discussions

### 4.1 Effect of low-temperature on rice biomass and yield

Rice spikelet fertility, filling and setting rate are most susceptible to temperature extremes mainly in the last week before and during the rice heading stage, and low-temperatures during the rice heading stage resulted in reduced rice biomass and yield (Lü et al., 2013; Ren et al., 2017). In the present study, it was found that rice yield of JN809 and J88 treated with low-temperature during heading stage was 27.6% and 21.4% lower than CK, which is consistent with previous studies (Ghadirnezhad and Fallah, 2014; Zeng et al., 2017). The reasons might be that low temperature stress directly affected microspore differentiation and development, resulting in the reduction of the number of microspore differentiation and the dysplasia of differentiated microspores, resulting in poor or unable to crack anther, lower fertilization rate, and ultimately led to spikelet abortion, decreased the percentage of grain filling and reduced kernels per panicle and yield (Farrell et al., 2006; Li et al., 2014; Jia et al., 2019). Furthermore, low-temperature stress also reduced photosynthetic rate, chlorophyll fluorescence and dry matter accumulation, resulting in lower yield (Siddik et al., 2019; Ai et al., 2023). Moreover, the reduction in N uptake under low-temperature treatment could likewise lead to a decrease in leaf area index and net photosynthetic rate, which in turn inhibited normal panicle development (Jia et al., 2019).

Different varieties have different abilities to tolerate low-temperature stress (Wainaina et al., 2015; Geraldine et al., 2020). Low temperature stress could cause a certain degree of harm to the antioxidant system in rice flag leaves. Under low-temperature stress, with the continuous increase of reactive oxygen species, the activity of protective enzymes in rice flag leaves such as superoxide dismutase (SOD), catalase (CAT), and peroxidase (POD) increased, in order to maintain the free radicals at an appropriate level and reduce the damage to cells by free radicals (Li et al., 2014; Han et al., 2023). However, when the temperature was too low, the activity of protective enzymes in rice leaves was affected, leading to excessive accumulation of peroxides and a rapid increase in membrane unsaturation. The lipid membranes were extremely prone to oxidation. This led to damage to rice photosynthesis and other functions, affecting the stable formation of final yield. Studies found that cold- tolerant varieties were more likely to induce antioxidant enzyme activity under low-temperature treatment than non-cold- tolerant varieties (Guo et al., 2006; Gazquez et al., 2018). At the same time, previous studies have shown that overexpressing antioxidant enzymes in transgenic plants can better resist low-temperature stress (Kornyeyev et al., 2003; Wang et al., 2005). In this study, the yield decline of low-temperature tolerant variety J88 after low-temperature stress treatment was significantly lower than that of JN809 variety. This may be because the low-temperature tolerant variety J88, with higher physiological adaptive capacity, secreted more antioxidant enzymes from the leaves after being subjected to low-temperature stress to resist the adversity stress, thus slowing down the effect of low-temperature stress on the development of rice pollen mother cells and glumes (Shimono et al., 2007), which resulted in a lower rate of rice empty shells and a smaller decrease in yield (Susanti et al., 2019). This phenomenon was confirmed by the significantly higher kernel number of J88 variety than that of JN809 variety in the present study. Ali et al. (2021) similarly found that low-temperature tolerant varieties showed less yield reduction after exposure to low-temperature stress.

### 4.2 Effect of low-temperature on N allocation

Nitrogen is one of the most important and active nutrient factors affecting the growth and yield of rice (Mi et al., 2017). Low-temperature cold damage interferes with plant physiological metabolic processes such as water metabolism, mineral nutrition, photosynthesis, respiration, and metabolism, and has a significant effect on their uptake of soil N (Lu et al., 2005; Zhao et al., 2015). Our results showed that the rhizosphere soil ^15^N contents of JN809 and J88 under low-temperature treatment were significantly lower than CK, while the bulk soil ^15^N contents were significantly higher than CK, respectively. These results indicate that low-temperature treatment reduced soil N uptake by rice root, which was mainly due to the weakened photosynthesis and reduced physiological metabolism of rice due to low-temperature, thus leading to lower nutrient demand of rice plants (Jia et al., 2019). Moreover, the total biomass of JN809 and J88 was lower under the low-temperature treatment than CK, respectively, which indicated that low-temperature could reduce the nutrient requirement of the rice plant. Rice yield is closely related to nitrogen absorption and utilization capacity of rice varieties (Fageria, 2003). In order to ensure normal ear development, it is necessary to maintain appropriate nitrogen content and reasonable balance of carbon and nitrogen metabolism during ear differentiation (Lawlor, 2002). Low-temperature stress leads to a reduction in the rate of N transport and a decrease in N accumulation in the rice panicle (Shimono et al., 2012). In this study, it was found that low-temperature stress during the rice heading stage led to a tendency for N accumulation in the bulk soil and stem and leaf parts, while the accumulation in the panicle was reduced (**Figure 4**). At rice harvest, the bulk soil ^15^N isotope contents of JN809 and J88 varieties treated at low-temperature stress were higher than CK, respectively, while the panicle ^15^N isotope contents were lower than CK, respectively. This suggests that low-temperature stress reduces N uptake by rice and affects N translocation to the rice panicle (Shimono et al., 2012; Liu et al., 2018). Jia et al. (2019) also found that low-temperature chilling reduced the ability of rice to absorb N and reduced rice N production by 4%-47%. The nitrogen absorption and utilization capacity of different rice varieties were different (Fageria, 2003). Low-temperature tolerant rice varieties can mitigate the impact of low-temperature stress on N absorption by rice. This study found that the ^15^N isotope content in both the aboveground and underground parts of the low-temperature tolerant variety J88 was higher than that of the conventional rice variety JN809. The reason may be that the low-temperature tolerant rice variety J88 had stronger physiological adaptability and genetic basis, which could maintain high growth activity and nutrient absorption capacity in low-temperature environments. At the same time, low-temperature tolerant rice varieties may also have better stress resistance mechanisms, which could alleviate the adverse effects of low-temperature on nutrient absorption and distribution by adjusting their internal physiological processes and metabolic pathways (Liu et al., 2015; Wu et al., 2017).

### 4.3 Effect of low-temperature on C allocation

Plant photosynthetic C is an important component of the atmosphere-plant-soil C cycle in terrestrial ecosystems (Schlesinger and Andrews, 2000). Plants convert CO_2_ into organic matter through the photosynthetic C assimilation pathway, and the resulting photosynthetic C is transported through the plant phloem and distributed to the plant-soil system. A portion of this photosynthetic C is imported into the below-ground part for root growth, while C is imported to the soil in the form of root deposits (Uchida et al., 2010). The other part is stored in the above-ground part of the plant. Low-temperature treatment affected photosynthetic C allocation in rice (Jia et al., 2019, Jia et al., 2022). In this study, we found that at the end of the low-temperature treatment, the soil ^13^C isotope contents of the low-temperature treated JN809 and J88 varieties were 26.7% and 113.3% higher than CK, while the rice panicle ^13^C isotope contents of JN809 and J88 were significantly lower than CK, respectively. This may be due to the low-temperature treatment slowing down the fertility process in rice. The proportion of photosynthetic C allocated to the root system and soil was high in the vegetative growth stage of the rice-soil system (Werth and Kuzyakov, 2010). The proportion of photosynthetic C allocated to the root system and soil tended to decrease as the reproductive period progressed (Deng et al., 2017; Zang et al., 2019), and photosynthetic C tended to be allocated more toward the panicle portion. In addition, the reduction in soil N uptake by rice plants under the low-temperature treatment led to a decrease in leaf area index and net photosynthetic rate, which inhibited normal panicle development (Jia et al., 2019), then likewise resulted in reduced photosynthetic C transport to the panicle in rice. The ^13^C isotope contents of rice stem, leaf and root of different varieties under low-temperature treatment during rice harvest were higher than CK, and the ^13^C isotope content of rice panicle of J809 variety was lower than CK. This could also indicate that the low-temperature treatment affected the photosynthetic C transport in rice, leading to a reduction in photosynthetic C accumulation in the panicle, which in turn affected the formation of rice yield.

Low-temperature tolerant rice varieties effectively mitigate the impact of low-temperature stress on the distribution of photosynthetic carbon within the rice-soil system. According to our research, during the grain filling stage, the ^13^C isotope partitioning ratios in the stems and leaves of the low-temperature tolerant variety J88 were lower than JN809. However, during the harvesting stage, the ^13^C isotope partitioning ratio in the rice panicles of J88 was higher than that of JN809 (**Figure 7**). This difference was likely attributed to the unique genetic traits of the low-temperature tolerant varieties, which allowed them to maintain robust growth activity and nutrient absorption even under low-temperature conditions (Liu et al., 2015). As this study was only carried out once for ^13^C isotope labelling prior to the low-temperature treatment, it could not reveal the transport pathways of the newly transformed photosynthetic C after the low-temperature treatment. Follow-up studies could be carried out several times after the end of the low-temperature treatment for ^13^C isotope labelling, which could provide more clarity on the allocation of photosynthetic C to rice by the low-temperature treatment.

### 4.4 Limitation

In this study, we primarily investigated the effects of low-temperature stress on the allocation of carbon (C) and nitrogen (N) in different rice varieties. However, we recognize that the regulatory mechanisms of C and N allocation under low-temperature stress are multifaceted. Among these, key metabolic enzymes play an indispensable role (Nunes-Nesi et al., 2010). For instance, sucrose phosphate synthase (SPS) and sucrose synthase (SUSY) are key enzymes in regulating carbohydrate metabolism, playing important roles in the synthesis and breakdown of sucrose within plants (Li J. et al., 2024). Sucrose, as the main form of C transport in plants, experiences changes in its metabolic levels under low-temperature stress, which directly affects the plant’s C allocation and biomass accumulation (Koch, 2004). In leaves, the activities of SPS and SUSY may be closely related to the transport and storage of photosynthetic products, thereby influencing the allocation of C to other organs, such as grains (Li J. et al., 2024). Additionally, enzymes related to N assimilation (such as glutamine synthetase and glutamate synthase) play a central role in the absorption, transformation, and transport of N (Dubey et al., 2021). The rational allocation of N is crucial for plant growth, development, and yield formation, especially during grain development, where the effective supply and allocation of N directly affect protein synthesis and grain quality (Fageria, 2014). Although we were unable to measure the activities of these metabolic enzymes and the levels of related metabolites in this study, future research could consider a more in-depth analysis of the activities of these enzymes under low-temperature stress and their correlation with biomass accumulation. This would help to provide a more comprehensive understanding of the regulatory mechanisms of C and N allocation in different rice varieties under low-temperature stress.

### 4.5 Implication and strategy

Low-temperature stress is one of the major limiting factors affecting stable rice production in high-latitude areas (e.g. northeast China). Delayed-type or obstructive-type cold damage seriously affects rice growth, development, and grain filling (Tao et al., 2013). Due to the low temperature stress reduced the absorption of N by rice root system and the transfer of nitrogen to rice panicles, which disrupted the transportation of photosynthetic C in rice. However, the low-temperature tolerant varieties could effectively alleviate the influence of low temperature stress on the distribution of photosynthetic C and N absorption in rice-soil system, thus reducing the adverse effects on the growth and development of rice panicles. By understanding the distribution and transformation of C and N in plants and soil under low-temperature stress, appropriate measures can be taken to cope with the adverse effects of climate change on rice. For example, rational fertilization and irrigation, application of plant growth regulators, adjusting the sowing date, and improving the stress resistance of rice (Huang et al., 2019; Wang et al., 2023; Yang et al., 2024). In addition, using modern biotechnology methods such as gene editing to cultivate low-temperature tolerant rice varieties and improve their stress resistance is also the most important measure to cope with climate change (Li K. et al., 2024).

## 5 Conclusions

Low-temperature stress during the rice heading stage reduced biomass and yield, mainly by decreasing the number of rice panicles and lowering the setting rate. This effect was primarily due to reduced N uptake by rice roots, decreased N translocation to the rice panicles, and disrupted photosynthetic C transport in rice under low-temperature treatment. In contrast, the low-temperature tolerance varieties effectively mitigated the impact of low-temperature stress on photosynthetic C distribution and N uptake within the rice-soil system, thereby reducing the adverse effects on panicle growth and development. Considering the limitations of the pot experiment, further field trials are needed to validate these findings and to better understand the mechanisms involved. This will help in developing more effective countermeasures for rice encountering low-temperature damage.

## Data Availability

The original contributions presented in the study are included in the article/**Supplementary Material**. Further inquiries can be directed to the corresponding author.
