# Supplementary material for: Effects of low-temperature stress during rice heading stage on carbon and nitrogen allocation in paddy eco-system of northeastern China
Source: Front Plant Sci. 2025 Mar 21;16:1484734. doi: 10.3389/fpls.2025.1484734 (PMC11968695; doi:10.3389/fpls.2025.1484734)
Supplement: Supplementary file 1 [file DataSheet1.docx]

**Supplementary tables and figures:**

Fig. S1 ^13^C and ^15^N labelling (A), artificial climate chamber (B), low-temperature stress treating of rice (C), and air temperature controlling in artificial climate chamber from 20:00 to 8:00 (D) at the experimental site.
